# Oligomerization of 3,5-Dimethyl Benzyl Alcohol Promoted by Clay: Experimental and Theoretical Study

**DOI:** 10.3390/molecules15118156

**Published:** 2010-11-11

**Authors:** José Antonio Morales-Serna, Luis E. López-Duran, Miguel Castro, Luis E. Sansores, Mikhail Zolotukhin, Manuel Salmón

**Affiliations:** 1 Instituto de Química de la Universidad Nacional Autónoma de México, Circuito Exterior, Ciudad Universitaria Coyoacán 04510, México, D.F., Mexico; E-Mail: salmon@unam.mx (M.S.); 2 Facultad de Química de la Universidad Nacional Autónoma de México, Circuito Interior, Ciudad Universitaria Coyoacán 04510, México, D.F., Mexico; E-Mail: castro@quetzal.pquim.unam.mx (M.C.); 3 Instituto de Investigaciones en Materiales, de la Universidad Nacional Autónoma de México, Circuito Exterior, Ciudad Universitaria Coyoacán 04510, México, D.F., Mexico; E-Mails: sansores@servidor.unam.mx (L.E.S.); zolotukhin@iim.unam.mx (M.Z.)

**Keywords:** montmorillonite clay, oligomerization, antrhacene, EPR, radical cation

## Abstract

Linear oligomerization of 3,5-dimethyl benzyl alcohol is induced by a montmorillonite clay (Tonsil Optimum Extra), producing 1,3,5,7-tetramethyl-9,10-dihydro-anthracene, which, by loss of protons results in the product 1,3,5,7-tetramethylanthracene. It was also found that the compounds 4-(3´,5´-dimethylbenzyl)-1,3,5,7-tetramethyl-9,10-dihydroanthracece and 4-(3´,5´-dimethylbenzyl)-1,3,5,7-tetra-methylanthracene were formed from 1,3,5,7-tetramethyl-9,10-dihydroanthracene. 1,3,5,7-Tetramethylanthryl radical cation was formed from 1,3,5,7-tetramethyl-9,10-dihydroanthracene; it was characterized by Electronic Paramagnetic Resonance (EPR). On the other hand, a theoretical analysis was performed, allowing the rationalization of the observed products and some of the key reaction steps.

## 1. Introduction

The development of general, catalytic and selective procedures for the oligomerization of benzyl alcohols is highly desirable and would constitute a broadly applicable set of transformations in organic synthesis. Considering the fact that a benzyl alcohol C-O bond is more reactive than that of an aliphatic alcohol, benzyl alcohol C-O activation is favoured in acid catalytic reactions due to the higher stability of intermediate formed [1]. Even though the oligomerization of alkenes catalyzed by montmorillonite has been developed for polymer syntheses of industrial interest [2], fewer advances have been made in the realm of selective oligomerization of benzyl alcohols. Since Pillali [3] established the first examples of controlling oligomerization to form anthracene, the use of this process has not been explored to achieve that goal. General strategies for selective benzyl alcohol C-O activation would open the door to a variety of useful, eagerly sought funtionalizations.

In this context, we considered the possibility of carrying out the oligomerizacion of benzyl alcohols in the presence of montmorillonite (Tonsil Optimum Extra) [4] to obtain fused aromatic hydrocarbons. These compounds, whose structures have photo and electrochemical [5] properties employed in optical devices [6], polymeric materials [7], and potential therapeutics [8] are commonly known as acenes [9]. Anthracene and its derivatives are some of the most important types of polycyclic aromatic compounds prepared by means of a Friedel-Crafts reaction [10], aromatic cyclodehydration [11], Lewis acid-induced Bradsher-type reaction from diarylmethanes [12], acid-promoted transannular cyclodehydration [13] and homologation mediated by metallacycles [14,15]. Thus, in the present article, we report an experimental and theoretical study of the linear oligomerization of 3,5-dimethyl benzyl alcohol using a montmorillonite as an efficient catalyst to form the 1,3,5,7-tetramethyl-anthracene (**4**). 

## 2. Results and Discussion

The procedure is based on the addition of Tonsil to a carbon disulfide solution of 3,5-dimethyl benzyl alcohol (**1**). At reflux 1,3,5,7-tetramethyl-9,10-dihydroanthracene (**2**) is formed (1%) within 15 hours. 

**Scheme 1:**
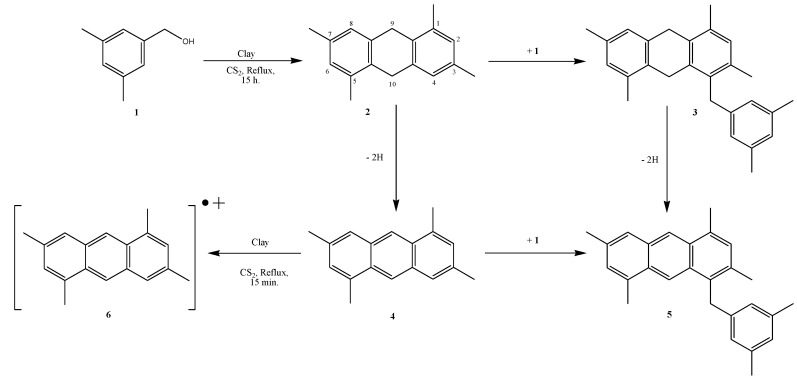
Oligomerization reaction.

This intermediate furnished the 4-(3´,5´-dimethylbenzyl)-1,3,5,7-tetramethyl-9,10-dihydro-anthracece (**3**, 0.23%) and 1,3,5,7-tetramethylanthracene (**4**, 85%). Both compounds **3** and **4** are direct precursors of 4-(3´,5´-dimethylbenzyl)-1,3,5,7-tetramethylanthracene (**5**), which was formed in 5% yield (Scheme 1).

**Figure 1:**
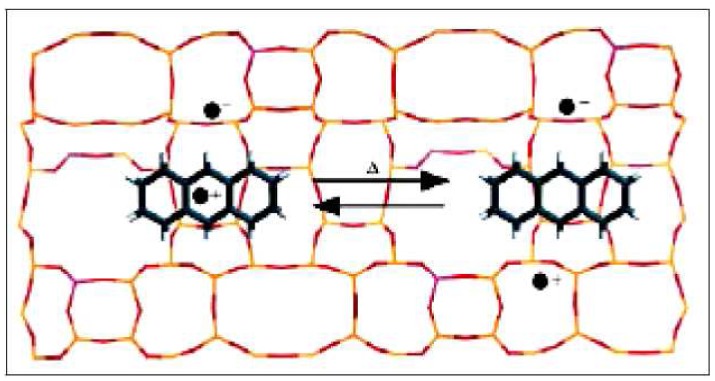
Schematic structure of radical cation **6 **inside a montmorillonite.

**Figure 2:**
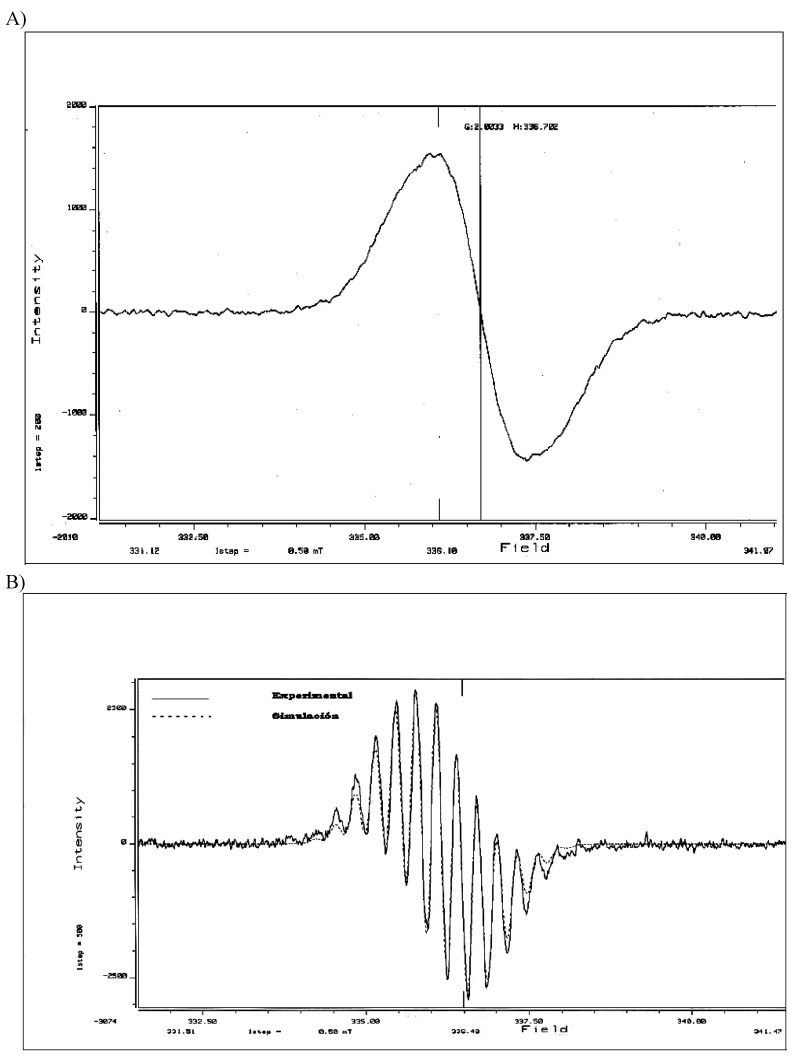
A) EPR spectrum corresponding to the clay containing the radical cation **6 **(298 K). B) EPR experimental and simulated spectra of clay containing the radical cation **6.**

**Figure 3:**
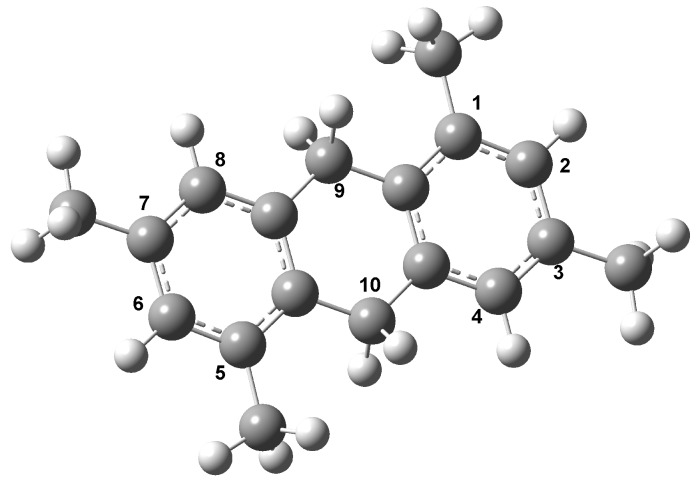
Structure of compound **2**.

Then 1,3,5,7-tetramethylanthracene (**4**) was treated with more Tonsil to give the radical cation **6**, which remained inside the clay and caused an intense red color in the solid material. Using EPR spectroscopy, we were able to establish and confirm the presence of the radical cation inside the clay (Figure 1). The EPR spectrum corresponding to the clay containing radical cation **6 **showed an isotropic signal persistent for two months, with hyperfine coupling interactions centered at *g* = 2.0033 corresponding to a typical organic free radical and surely indicating a doublet state (Figure 2 and Figure 3). The hyperfine coupling constant (hfcc) values for that signal were: *a*_H_ = 0.107 mT, with a line width of ∆*H*_p-p _= 18.39 mT. These observations can be rationalized as the capacity of the clay to generate and stabilize the radical cation, due to the presence of Al^+3^ in the laminar structure of the aluminosilicate. 

**Figure 4:**
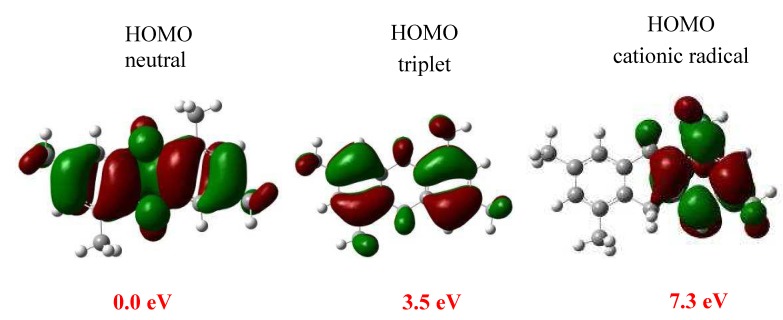
Shape of molecular orbitals.

From our theoretical results, we assume that the 3,5-dimethylbenzyl alcohol undergoes a reaction with the clay inducing the formation of radical species, doublets or triplets and even the formation of cationic doublets, which is due to the high acidic conditions; overall, these radical states are expected to be highly reactive. Under the experimental conditions described above lines, the first product **2** is the one that is obtained (Figure 3). The neutral GS of 1,3,5,7-tetramethyl-9,10-dihydroanthracene (**2**) as well as its radical (triplet) and cationic radical (doublet) states were also characterized. The structures are displayed in Figure 4.

**Figure 5:**
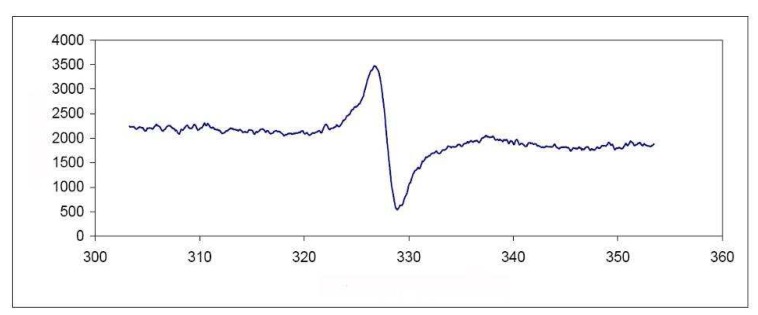
EPR spectrum of the reaction.

The energy indicates that the triplet and radical cationic states are localized respectively at 3.5 and 7.3 eV above of the neutral **2**. Our theoretical results indicate that the cationic radical state is the one of highest energy, whereas the experimental results of the electron paramagnetic resonance (EPR), suggest that the radical species play an important role on the oligomerization reaction. The EPR spectrum (Figure 5) corresponding to the reaction showed a signal centered at *g* = 2.0017 (H = 328.30), with a line width of ∆*H*_p-p _= 18.39 mT (183.9 G). 

In Table 1 the charge distribution of the cationic radical state is displayed, revealing that the region defined by the C9 and C10 atoms has the most favorable reactive position to former **4**. The analyses of the contour-plot for the HOMO orbital indicate that it is widely distributed at the whole of the neutral molecular structure **2**, avoiding the clear identification of the preferred reactivity sites (see Figure 4). Additionally, for the triplet radical **2**, located at 3.5 eV over the neutral GS, the HOMO analysis shows a different result. The strong contributions on C2 and C4 suggest that these carbons are the most reactive sites in the formation of **3**. Consequently, these features, altogether with the high spin density, of ↑0.77 e, on the C4 atom, support the appearance of product **3 **as intermediate in all the processes. Thus, on the early steps the oligomerization process seems to follow an orbital type control, with the spin effects playing an important role. 

**Table 1:** Charge distribution on the carbon atoms for **2** compound.

	C2	C4	C6	C8	C9	C10
**Neutral**	–0.167	–0.176	–0.167	–0.176	–0.313	–0.313
**Triplet**	–0.148	–0.181	–0.166	–0.175	–0.308	–0.301
**Radical cationic**	–0.136	–0.159	–0.136	–0.159	–0.349	–0.349

**Figure 6:**
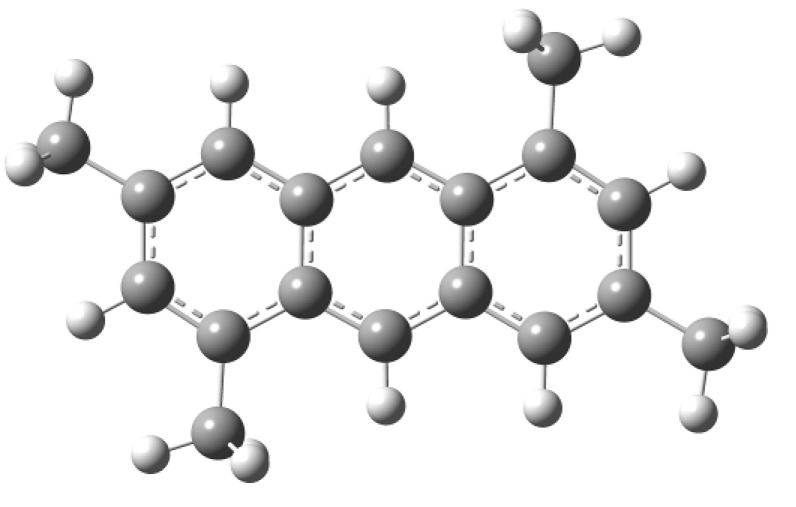
Structure of compound **4**.

Starting from the reactive structure **2** and considering the effects of the clay, two hydrogen are removed leading to the appearance of 1,3,5,7-tetramethylanthracene (**4**, Figure 6) which is more stable than **2**. 

**Figure 7:**
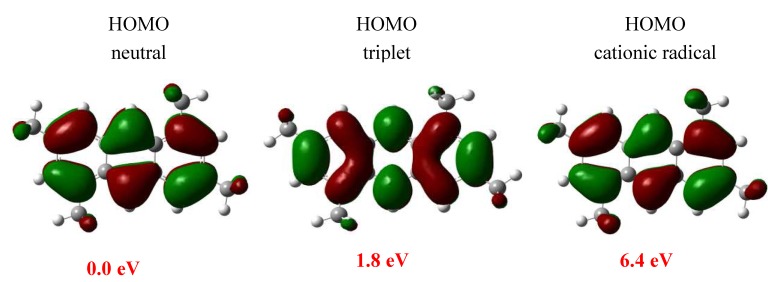
Shape of molecular orbitals.

The energy stabilization of the neutral, radical (triplet) and radical cationic GS structures also shows that the neutral compound is more stable that the triplet and radical cationic species. The neutral, triplet and cationic radical states of **4** are displayed in Figure 7. The triplet state, localized at 1.8 eV above of the neutral state, having a high spin density of ↑0.59 e over the C9 and C10 atoms whereas ↑0.22 e is presented on the C8 atom. The cationic radical localized 4.6 eV above the triplet state have the same behavior. The charge of **4** (see Table 2), indicates that there are no significant differences between C4, C8, C9 and C10 such reactive sites. The experimental results reveal that the region defined by the C4 or C8 has the most favorable reactive position to form **5**.

**Table 2:** Charge distribution on the carbon atoms for **4** compound.

	C2	C4	C6	C8	C9	C10
Neutral	–0.180	–0.183	–0.180	–0.183	–0.226	–0.226
Triplet	–0.172	–0.201	–0.172	–0.201	–0.220	–0.220
radical cationic	–0.149	–0.160	–0.149	–0.160	–0.168	–0.168

The chemical potential, global hardness and the Fukui functions were calculated. It should be noted that no optimization was done either for the anion or the cation since all of them were defined from the vertical ionization potential and the vertical electron affinity. The chemical potential and global charges for the four molecules are given in Table 3. It should be noted that while the chemical potential of the four molecules is almost the same, the global hardness of molecule **2** is much bigger than that of molecule **4**, and that of molecule **3** almost doubles that of **5**. This indicates that **4** and **5** are more reactive than **2** and **3**, respectively, which is in agreement with the yields observed experimentally (Scheme 1).

**Table 3:** Chemical potential (μ) and Hardness (η) in eV for compounds under study.

Compound	μ	η
**2**	–0.119	0.108
**4**	–0.110	0.043
**3**	–0.099	0.082
**5**	–0.110	0.042

To complement the charge analysis and find the active sites of the molecules, we have calculated the Fukui functions. f^–^ indicates the activity for electrophilic attack, f^+^ for a nucleophilic attack and f^0^ for a radical attack (Table 4 and Table 5). 

**Table 4:** Fukui indexes f^+^, f– and f^0^ for selected atoms (for notation refer to Scheme 1).

Atom	2			4		
	f^–^	f^+^	f^0^	f^–^	f^+^	f^0^
C1	0.058	0.025	0.042	0.013	0.021	0.017
C2	–0.008	0.012	0.002	0.029	0.023	0.026
C4	0.090	0.046	0.068	0.033	0.034	0.033
C9	–0.002	–0.010	–0.001	0.049	0.050	0.049

**Table 5:** Fukui indexes f^+^, f^–^ and f^0^ for selected atoms. (For notation refer to compound **3**).

Atom	3			6		
	f^–^	f^+^	f^0^	f^–^	f^+^	f^0^
C1	0.011	0.017	0.014	0.012	0.022	0.017
C2	0.013	0.005	0.009	0.024	0.020	0.022
C3	0.010	0.007	0.009	0.018	–0.004	0.011
C4	0.007	0.020	0.013	0.023	0.033	0.028
C5	0.007	0.018	0.013	0.012	0.018	0.015
C6	0.021	0.008	0.015	0.027	0.021	0.024
C7	0.005	0.002	0.003	0.006	0.000	0.003
C8	0.018	0.032	0.025	0.029	0.031	0.030
C9	–0.010	–0.009	–0.010	0.045	0.047	0.046
C10	0.007	–0.005	–0.006	0.050	0.054	0.052
C42	0.023	0.021	0.023	0.001	–0.003	–0.001
C43	0.010	0.028	0.019	–0.003	–0.005	–0.004
C48	0.023	0.008	0.015	0.009	0.005	0.007

**Figure 8:**
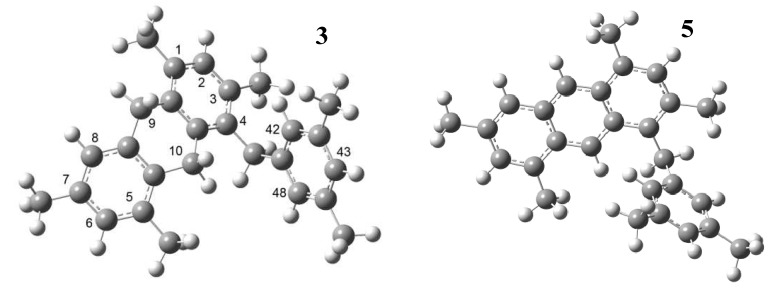
Structures of compounds **3** and **5**.

Molecule **2 **is more active towards an electrophilic attack through atoms C4 and C1. The same atoms are active for a radical attack. Molecule **4** has the same activity for any of the three attacks and sites C9 and C4 are the more active ones. Molecule **3** is much less active than **2** and **4**; the active sites for an electrophilic attack would be C48 and C42, which are located in the new branch, and C6 (Figure 8). 

It should be noticed that the activity of site C8 is strongly diminished in comparison to molecule **2**. The active sites for a nucleophilic attack are C8 and C43, while for a radical attack they are C8 and C42. In molecule **5**, the active sites for any of the three types of attack are C9 and C10, although sites C8 and C6 are also active to a lower degree.

## 3. Experimental

### 3.1. General

All solvents and reagents were commercial grade (Aldrich). Yields refer to the chromatographically and spectroscopically (^1^H- and ^13^C-NMR) homogeneous materials, unless otherwise stated. Reactions were monitored by TLC carried out on 0.25 mm E. Merck silica gel plates. Developed TLC plates were visualized under a short-wave UV lamp and by heating plates that were dipped in Ce(SO_4_)_3_. Flash column chromatography (FCC) was performed using flash silica gel (32-63 μm) and employed a solvent polarity correlated with TLC mobility. Melting points were determined with a Fisher-Johns apparatus, and are uncorrected. NMR experiments were conducted on a Varian 400 MHz instrument using CDCl_3_ (99.9% D) as solvent. Chemical shifts are in ppm with respect to TMS (tetramethylsilane). Infrared spectra were recorded as pellet/KBr with a Fourier transform (FTIR) Nicolet FT-IR 750 spectrometer. Mass spectra were recorded on Jeol JS102 high-resolution mass spectrometer. 

Tonsil Actisil FF (TAFF), is a commercial Mexican bentonitic clay, easily available from Tonsil Mexicana S.A. de C.V., at US$ 1.30/kg. Examined with x-ray fluorescence, the clay proved to have the following composition (in percent): SiO_2_, 74.5; Al_2_O_3_, 9.3; MgO, 0.4; Fe_2_O_3_, 1.3; CaO, 4.0; K_2_O, 0.4; TiO_2_, 0.4; H_2_O, 9.7. When x-ray thermodiffractograms were run, the laminar structure was found to be unstable above 150 °C. Quartz and cristobalite are also important components on the clay composition as observed by x-ray diffraction. The corresponding BET surface area was 198.718 m^2^g^-1^ and the pore volume and pore average diameter were 32.04 × 10^-2^ cm3 g^-1^ and 77.8 Å respectively [16].

The EPR measurements of radical cations were made on a flat cell at room temperature, with a Jeol JES-TE300 spectrometer operating at X-Band mode, at a modulation frequency of 100 kHz. The spectra were simulated using the program ESPRIT-382, v1.916.

### 3.2. Benzyl Alcohol Oligomerization with Tonsil

A suspension of 3,5-dimethylbenzyl alcohol (**1**, 1.5 g), in carbon disulfide (20 mL) and Tonsil (0.375 g) was stirred and refluxed until the substrates disappeared (15 h). The reaction advance was monitored by TLC. The clay was removed by filtration through Celite and washed with portions of ethyl acetate (3 × 50 mL). The organic layer was concentrated under vacuum and the resulting crude was purified by flash column chromatography on silica gel with a hexane-ethyl acetate gradient (9:1→1:9) to give compounds **2**–**5**.

*1,3,5,7-tetramethyl-9,10-dihydroanthracene* (**2**). Yellow oil (1%). ^1^H-NMR: δ 6.97 (4H, d, *J* = 1.5 Hz), 6.88 (4H, d, *J* = 1.5 Hz), 3.84 (4H, s), 2.34 (s, 6H), 2.29 (s, 6H); ^13^C-NMR: δ 134.8, 131.4, 128.4, 126.3, 32.1, 20.8, 19.5; IR (KBr pellet): 2924, 2859, 1603, 1460, 1375, 845; HRMS (FAB) calcd for C_18_H_20_ 236.3114, found 236.3112.

*4-(3´,5´-dimethylbenzyl)-1,3,5,7-tetramethyl-9,10-dihydro-anthracece* (**3**). Yellow oil (0.23%). ^1^H-NMR: δ 7.11 (1H, brs), 6.83 (3H, m), 6.62 (2H, brs), 3.92 (4H, m), 2.32 (s, 3H), 2.27 (s, 3H), 2.214 (s, 6H); ^13^C-NMR: δ 142.8, 138.1, 135.7 135.2, 133.3, 131.0, 129.2, 128.8, 128.4, 127.8, 127.6, 127.4, 127.3, 126.1, 125.0, 34.7, 30.5, 24.8, 24.0, 19.0, 18.9; IR (KBr pellet): 2924, 2858, 1603, 1460, 1379, 1379, 849; HRMS (FAB) calcd for C_27_H_30_ 354.5271, found 354.5268.

*1,3,5,7-tetramethylanthracene* (**4**). Yellow solid (85%). m.p. 164-165 °C; ^1^H-NMR: δ 8.35 (2H, brs), 7.63 (2H, brs), 7.13 (2H, brs), 2.75 (s, 6H), 2.49 (s, 6H); ^13^C-NMR: δ 134.1, 133.7, 131.7, 129.9, 128.5, 125.3, 122.1, 21.9, 19.5; IR (KBr pellet): 3051, 2976, 2943, 2920, 2860, 1635, 1466, 1439, 1377, 1360, 896, 881; HRMS (FAB) calcd for C_18_H_18_ 234.3355, found 234.3348.

*4-(3´,5´-dimethylbenzyl)-1,3,5,7-tetramethylanthracene* (**5**). Yellow oil (5%). ^1^H-NMR: δ 8.57 (1H, brs), 8.36 (1H, brs), 7.6 (1H, brs), 7.24 (1H, brs), 7.18 (1H, brs), 7.07 (1H, brs), 6.8 (2H, brs), 6.74 (1H, brs), 4.49 (2H, brs), 2.76 (s, 3H), 2.61 (s, 3H), 2.5 (s, 3H), 2.46 (s, 3H), 2.18 (s, 6H); ^13^C-NMR: δ 141, 137.8, 134.2, 132.4, 131.6, 131, 130.8, 130.7, 129.7, 129.5, 128.4, 127.4, 126, 124.8, 122.4, 119.9, 34.6, 21.8, 21.3, 20.6, 19.6, 19.4; IR (KBr pellet: 3010, 2921, 2856, 1616, 1635, 1477, 1439, 1373, 1073, 1033, 877, 841; HRMS (FAB) calcd for C_27_H_28_ 352.5112, found 352.5110.

### 3.3. Formation of 1,3,5,7-tetramethylanthryl Radical Cation (*6*)

Tonsil (100 mg) was added to a solution of 1,3,5,7-tetramethylanthracene **4** (30 mg) in CS_2_ (5 mL) at room temperature. Then, the reaction mixture was stirred for 15 minutes at reflux observing a red color. After removal of the CS_2_, the red solid residue was analyzed by EPR.

### 3.4. Theoretical Study

The geometry and electronic structure of the ground states (GS) of all species, reactants and products, as well of those of the intermediate states, were studied by means of *all-electron* calculations made with the B3LYP functional and 6-31G (d, p) orbital basis sets. The calculations were carried out with the Gaussian 03 quantum chemistry software [17]. The optimised structures were confirmed as local minima, by estimating their normal vibrations. Charge distributions, through Mulliken population analysis, and spin densities were determined for the equilibrium geometries. The structures and the molecular orbitals (MO) were visualised with the Gauss View package, coupled to Gaussian-03. In particular, the frontier MOs, so-called highest occupied molecular orbital (HOMO) and lowest unoccupied molecular orbital (LUMO), and charge distributions, are very useful parameters that allow the characterisation of some key electronic properties of these species, which are responsible of the observed oligomerization pathway processes.

In the same context, DFT, based on the Hohenberg-Khon theorems, has proven an important tool for several chemical concepts and ideas on reactivity. The electronic chemical potential, μ [18], global hardness, η [19], and the so-called Fukui functions [20], f_x_^-^, f_x_^+^ and f_x_^0^, have been widely used in the rationalization of chemical processes.

Well known approximations to these parameters [20,21] are given by:

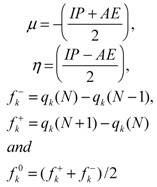

where IP is the ionization potential, AE is the electron affinity, q_k_(N) is the charge at the k atom in the molecule with N electrons. IP and AE can also be defined based on orbitals, on the basis of Koopmans’ theorem as IP = −E_HOMO_ and AE = −E_LUMO_, where E_HOMO_ and E_LUMO_ are the energies of the highest occupied molecular orbital (HOMO) and lowest unoccupied molecular orbital (LUMO), respectively.

## 4. Conclusions

The oligomerization of 3,5-dimethylbenzyl alcohol is induced by a montmorillonite clay, producing 1,3,5,7-tetramethylanthracene (**4)**. The experimental results allowed us to establish the importance of a radical cation as key intermediate in the formation of the anthracene. In the same context, the theoretical analysis helped to rationalize how the early steps the oligomerization process seems to follow an orbital type control. We are currently exploring the synthetic potential of this protocol for the construction of other polycyclic aromatic compounds.
